# Ubiquitin-Specific Peptidase 10 Protects Against Hepatic Ischaemic/Reperfusion Injury via TAK1 Signalling

**DOI:** 10.3389/fimmu.2020.506275

**Published:** 2020-09-29

**Authors:** Zhou Jiangqiao, Wang Tianyu, Chen Zhongbao, Zhang Long, Zou Jilin, Ma Xiaoxiong, Qiu Tao

**Affiliations:** Department of Organ Transplantation, Renmin Hospital of Wuhan University, Wuhan University, Wuhan, China

**Keywords:** USP10, ischaemic/reperfusion injury, liver, ubiquitination, TAK1

## Abstract

Ubiquitin-specific peptidase 10 (USP10) protein is a deubiquitination enzyme involved in many important biological processes. However, the function of USP10 in hepatic ischaemic/reperfusion (I/R) injury remains unknown. The aim of this study was to explore the role of USP10 in hepatic I/R injury. USP10 Heterozygote mice and primary hepatocytes were used to construct hepatic I/R models. The effect of USP10 on hepatic I/R injury was examined via pathological and molecular analyses. Our results indicated that USP10 was significantly downregulated in the livers of mice after hepatic I/R injury and in hepatocytes subjected to hypoxia/reoxygenation stimulation. USP10 Heterozygote mice exhibited exacerbated hepatic I/R injury, as evidenced by enhanced liver inflammation via the NF-κB signalling pathway and increased hepatocyte apoptosis. Additionally, USP10 overexpression inhibited hepatocyte inflammation and apoptosis in hepatic I/R injury *in vitro* and *in vivo*. Mechanistically, our study demonstrated that USP10 knockdown exerted its detrimental effects on hepatic I/R injury by inducing activation of the transforming growth factor β-activated kinase 1 (TAK1)-JNK/p38 signalling pathways. TAK1 was required for USP10 function in hepatic I/R injury as TAK1 inhibition abolished USP10 function *in vitro*. In conclusion, our study demonstrated that USP10 plays a protective role in hepatic I/R injury by inhibiting the activation of the TAK1-JNK/p38 signalling pathways. Modulation of USP10/TAK1 might be a promising strategy to prevent this pathological process.

## Introduction

Liver ischaemia reperfusion (I/R) injury is a common pathophysiological process during liver transplantation. Ischaemia reperfusion injury often leads to liver dysfunction and irreversible liver non-function, which is also the main factor causing acute and chronic rejection reactions in liver transplantation (1). As the marginal donor liver is very sensitive to ischaemic injury, I/R injury also limits the use of marginal donor liver (2).

During the period of liver ischaemia, danger-associated molecular patterns (DAMPs) activate Kupffer cells, neutrophils, and other immune cells, which then release inflammatory cytokines and chemokines (3). Inflammatory response cascades are stimulated, leading to further apoptosis and necrosis of liver cells. Therefore, the inflammatory response and apoptosis are key steps in liver I/R injury (4). It is particularly important to determine the regulatory mechanisms of inflammation and apoptosis to prevent and treat I/R injury.

Ubiquitin-specific peptidase 10 (USP10) is a member of the ubiquitin-specific peptidase family of cysteine proteases that cleaves ubiquitin from ubiquitin-conjugated protein substrates (5). USP10 is involved in environmental stress responses, tumour growth, inflammation, and cellular metabolism (6). In a non-alcoholic fatty liver disease model, USP10 inhibits hepatic steatosis, insulin resistance, and the inflammatory response by interacting with SIRT6 and inhibiting its ubiquitination and degradation (6), and USP10 inhibition also abolishes Mirt2 (long non-coding RNA myocardial infarction associated transcript 2) overexpression-induced suppression of glucose production and lipogenesis in hepatocytes (7). siRNA-mediated knockdown of USP10 and treatment with the USP10 inhibitor has been shown to effectively attenuate curcumin-induced paraptosis, a type of programmed cell death accompanied by dilation of mitochondria and/or the endoplasmic reticulum (8). In addition, stability and localisation of p53 is essential for tumour suppressor functions, while as a new regulator of p53 in the DNA damage response and tumour development, USP10 can regulate tumour development, including hepatocellular cancer (9–13). However, the pathophysiology of USP10 in hepatic ischaemic reperfusion has not been fully validated.

To date, the study revealed that USP10 alleviates hepatic I/R injury by inhibiting hepatocyte inflammation and apoptosis via the transforming growth factor β -activated kinase 1 (TAK1)-JNK/p38 signalling pathway in various ways. USP10/TAK1 may represent a druggable target for hepatic I/R injury.

## Materials and Methods

### Reagents

Antibodies against USP10 (8510), p-Ikkβ (2694), p-p65 (3033), p65 (8242), IkBα (4814), Bax (2772), Bcl2 (3498), Bax (2772), p-ERK (4370), ERK (4695), p-JNK (4668), JNK (9252), p-p38 (4511), p38 (8690), TAK1 (5206), p-ASK1 (3765), C-Caspase3 (9664), and GAPDH (2118) were purchased from Cell Signalling Technology. Antibodies against Ikkβ(A0714) and ASK1(A6274) were purchased from Abclonal. Antibodies against p-TAK1 were purchased from Abcam (ab192443). Antibodies against Ly6g were purchased from BD Biosciences (551459). Goat anti-mouse (115-035-003) and goat anti-rabbit (111-035-003) secondary antibodies were purchased from Jackson ImmunoResearch. Foetal calf serum was obtained from HyClone. Cell culture reagents and all other reagents were obtained from Sigma.

#### Animal Models and Procedures

USP10 heterozygote mice (USP10-HZ) and wild type littermates (WT) mice (male, 8 weeks old, 25–27 g) were obtained from the Animal Experiment Center of Wuhan University. We conducted experiments following the National Institutes of Health Guide for the care and use of laboratory animals. Mice were kept in an air-filtered, temperature-controlled (22–24°C), humidity-controlled (40–70%), and light-controlled room and were permitted free access to a standard diet.

#### Mouse Hepatic I/R Injury Model

The mouse hepatic I/R injury model was constructed according to the classical method with some small modifications (4, 14, 15). Mice underwent fasting for 12 h before surgery and were given free access to drinking water. The mice were anaesthetized with 3% pentobarbital sodium before the operation, their limbs were fixed in a horizontal position, and the hair of the abdominal area was shaved. The surgical area was disinfected with 10% iodine and 75% ethanol.

A ventral midline incision was made in the abdomen to expose the hepatic pedicles of the left and middle lobes of the liver. The portal vein and hepatic artery of the middle and left lobes were blocked with non-invasive vascular clamps, resulting in approximately 70% hepatic ischaemia to prevent severe mesenteric vein congestion. After 0.5 min, compared with the non-blocked right lobe, the blocked lobes turned white, indicating successful blood flow blockage. Then, the ischaemia was initiated and maintained for 60 min. No hepatic blood flow blockage was performed in sham group. After ischaemia, the vascular clamps were removed, and hepatic blood flow was restored. After the abdominal cavity was closed and sutured, the postoperative mice were placed in a clean cage and housed separately for observation.

##### Sampling

Mice were taken from the Sham operation group (Sham Group) and I/R group at the indicated time after surgery and anaesthetized with 3% pentobarbital sodium. Then, 1 ml of blood was taken from the orbital venous plexus and serum was isolated. At the same time, the middle hepatic lobes were collected and frozen in liquid nitrogen. The left hepatic lobe was fixed in a 10% formalin medium for 24 h, dehydrated, embedded, and prepared into paraffin sections.

### Isolation and Culture of Primary Hepatocytes

Primary hepatocytes were isolated from 6 to 8 week-old male mice as described previously (14–16). After anaesthesia, the mouse abdominal cavity was opened, and a needle was used to puncture the portal vein with 0.5% type IV collagenase (catalogue no. 17104-019). When the liver was digested completely, the liver was excised, minced and filtered through a 70-μm cell strainer (catalogue no. 352350; Falcon, BD Biosciences) to obtain primary hepatocytes. The cells were cultured in Dulbecco’s Modified Eagle’s Medium (DMEM) (catalogue no. 11965092; Invitrogen) supplemented with 10% FBS in plates coated with rat tail collagen at 37°C with 5% CO_2_.

For adenovirus infection, the primary hepatocytes were infected with indicated virus for 6 h. Twenty-four hours after viral infection, infected cells were selected with 4 μg/ml puromycin for additional 24 h cell culture, and then tested by western blot.

### *In vitro* Hhypoxic/Reoxygenation of Hepatocytes

To establish an I/R model *in vitro*, the primary hepatocytes were divided into a control group and an H/R group. The hepatocytes in the control Group were cultured in complete DMEM medium at 37°C with 5% CO_2_. For hepatocytes in the H/R group, the medium was replaced with serum-free DMEM medium equilibrated with 5% CO_2_ and 90% N_2_ and placed into a modular incubator chamber (Biospherix, Lacona, NY, United States) that was flushed with the same gas mixture. After 6 h, cells were incubated under normoxic conditions (air/5% CO_2_) for the 6h. The cells were then collected for further analysis.

The specific TAK1 inhibitor 5Z-7-oxozeaenol (5Z-7-ox; O9890-1MG; Sigma, St. Louis, MO, United States; 2.5 μM) was administered 30 min prior to hypoxia/reoxygenation (H/R) to primary hepatocytes isolated from USP10-HZ and WT mice. The same volume of dimethyl sulphoxide was used as the vehicle control.

### Plasmid and Adenovirus Constructs

For Adenovirus USP10 (AdUSP10), the sequence of USP10 gene was cloned into the adenovirus vector pENTR-CMV-ATG-flag-T2A-GFP to get the pENTR-CMV-m-USP10-flag-T2A-GFP. The primer sequences were as following: forward: F: GGCTAGCGATATCGGATCCGCCACCATGGCCCTCCACAA CCCACAG; R: CGTCCTTGTAATCACTAGTCAGCAGGTCCA CGCGGCGGT. pENTR-CMV-m-USP10-flag-T2A-GFP was recombined with the adenovirus plasmid pAd/PL-DEST to produce pAd-CMV-m-USP10-flag-T2A-GFP (AdUSP10).

For domain negative TAK1 (DnTAK1), the TAK1 whole cDNA sequence was cloned into the above adenovirus plasmid to produce the recombinant plasmid using the primers, F: GGCTAGCGATATCGGATCCGCCACCATGTCGACAGCCTC CGCCGCC; R: CGTCCTTGTAATCACTAGTTGAAGTGCCTT GTCGTTTCTGC. Domain negative TAK1 was produced at 63rd amino acid site changing from Lys to Asn using the primers F:GATGTCGCTATTAACCAGATAGAAAGTG;R: CACTTTCTATCTGGTTAATAGCGACATC. DnTAK1 plasmid was combined with adenovirus plasmid pAd/PL-DEST by Gateway to obtain DnTAK1 recombinant plasmid.

The recombinant adenovirus plasmid was linearised by *Pac*I enzyme, extracted by phenolic chloroform and transfected into 293A cells. The virus titre was determined by TCID50 method. The Ad-GFP particles without USP10 gene sequence was produced as the control adenovirus.

### Adenovirus-Mediated USP10 Overexpression Mice

The AdUSP10 particles (5 × 10^9^pfu per mouse) were injected into WT mice via tail vein to upregulate USP10 in hepatic tissue in mice. The control group was injected with AdGFP particles. The mice hepatic I/R model was constructed as described above post two days injection.

### Liver Function Analysis

Serum alanine aminotransferase (ALT) and serum aspartate aminotransferase (AST) levels in the serum were detected with an ADVIA 2400 biochemical analyser (Siemens, Tarrytown, NY, United States).

### Pathological Analysis

#### HE Staining

Liver sections (5 μm) were stained with H&E to analyse necrotic areas using Image Pro Plus software. The percentage of necrotic area in the total area of the tissue section was quantified blindly in more than five fields for each mouse. A pathologist who was blinded to the experimental protocol provided morphological assessments.

#### Terminal Deoxynucleotidyl Transferase dUTP Nick end Labelling Staining

To detect apoptosis induced by ischaemic reperfusion, we used an *in situ* apoptosis detection kit (ApopTag^®^ Plus *In Situ* Apoptosis Fluorescein Detection Kit, S7111; Millipore). Terminal deoxynucleotidyl transferase dUTP nick end labelling (TUNEL) assays were performed according to the manufacturer’s instructions.

Images were obtained under a fluorescence microscope (Olympus DX51). Transferase dUTP Nick end Labelling-positive cells per field of the tissue sections were quantified using Image-Pro Plus (version 6.0).

#### Immunohistochemistry

Paraffin liver sections were dewaxed to hydration, and C-Caspase-3 immunohistochemical staining was performed. Mouse liver sections were first sealed with BSA and repaired with EDTA at high temperature for 20 min. After that, the primary antibody (CST, 9664, United States) was incubated at 4°C overnight and rewarmed at 37°C for 30 min. After washing with PBS, the secondary antibody was incubated (Zhongshanjinqiao, PV9001, China).

#### Immunofluorescence

The infiltration of inflammatory cells into the liver sections was detected via immunofluorescence staining by applying CD11b or Ly6g antibody. After incubation with primary antibody overnight at 4°C, the liver sections were incubated with secondary antibody. Nuclei were labelled with DAPI. Immunofluorescence images were captured and analysed using Image-Pro Plus (version 6.0).

### Western Blotting

Protein samples of the same mass were added to the loading buffer and separated by 10% SDS-PAGE. After electrophoresis, the protein was transferred to a PVDF membrane. After the membrane was blocked with 5% skim milk powder at room temperature for about 1 h, the primary antibody was added and incubated overnight at 4°C. The secondary antibodies of the corresponding species were added and incubated at room temperature for 1 h. The blots were developed using an enhanced chemiluminescence system, and the results were captured on a light-sensitive imaging film. The signal was collected using a gel imaging system (ChemiDoc XRS+). Protein expression levels were quantified using Image J software.

### Real-Time Reverse Transcription-PCR

Tissues or cells were cleaved by Trizol according to the manufacturer’s instructions. Then, RNA was extracted and reverse transcribed into cDNA using Transcriptor First Strand cDNA Synthesis Kit. Target gene primers were designed, real-time reverse transcription-PCR (qRT-PCR) was performed with cDNA as template, and the relative mRNA expression levels of each gene was analysed with β-actin as an internal reference. Reaction conditions were set as follows: incubation at 95°C for 10 min, followed by 40 cycles of 95°C for 10 s and 60°C for 1 min. The relative mRNA expression levels were calculated using the 2^–ΔΔC^t method and were normalised against β-actin. The primers for each gene are listed in Table 1.

**TABLE 1:** Primer information.

Gene	Forward/Reforward	Sequence
USP10	F	GCTTGCCCTCCGATGTATCA
	R	TGGGCGGATATCTCTCACGA
IL6	F	TAGTCCTTCCTACCCCAATTTCC
	R	TTGGTCCTTAGCCACTCCTTC
IL1β	F	CCGTGGACCTTCCAGGATGA
	R	GGGAACGTCACACACCAGCA
Ccl2	F	TACAAGAGGATCACCAGCAGC
	R	ACCTTAGGGCAGATGCAGTT
TNF-α	F	CATCTTCTCAAAATTCGAGTGACAA
	R	TGGGAGTAGACAAGGTACAACCC
Bcl2	F	TGGTGGACAACATCGCCCTGTG
	R	GGTCGCATGCTGGGGCCATATA
Bax	F	TGAGCGAGTGTCTCCGGCGAAT
	R	GCACTTTAGTGCACAGGGCCTTG
Bad	F	CCAGAGTTTGAGCCGAGTGAGCA
	R	ATAGCCCCTGCGCCTCCATGAT
β-actin	F	GTGACGTTGACATCCGTAAAGA
	R	GCCGGACTCATCGTACTCC

### Statistical Analysis

All data are presented as mean ± SD. We used SPSS 19.0 software for all statistical analyses. Statistical differences among more than two groups were compared using one-way ANOVA, followed by Bonferroni analysis (for data meeting homogeneity of variance) or Tamhane’s T2 analysis (for data demonstrating heteroscedasticity). Statistical differences between two groups were compared using a two-tailed Student’s *t*-test. *P* < 0.05 was considered significant.

## Results

### USP10 Expression Was Decreased in the Hepatic I/R Process and Knockdown of USP10 Expression Exaggerated Hepatic Injury in a Mouse I/R Model

**FIGURE 1:**
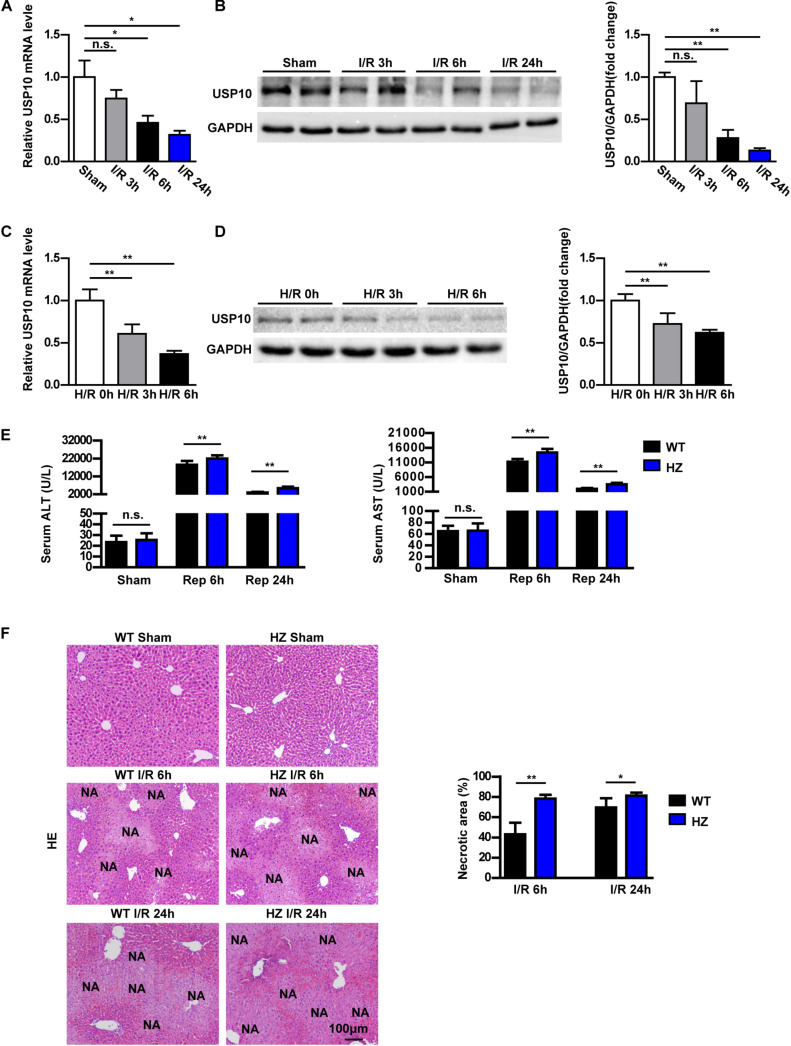
USP10 expression is downregulated in hepatic I/R models and USP10 knockdown exaggerates hepatic injury in an I/R model. **(A)** USP10 mRNA expression in WT livers at indicated time points after hepatic I/R injury (*n* = 6 per group). **(B)** USP10 protein expression in WT livers at indicated time points after hepatic I/R injury (*n* = 3 per group). **(C,D)** USP10 mRNA and protein expression in primary hepatocytes at indicated time points post H/R. **(E)** The ALT and AST serum levels in USP10-HZ and WT mice at 6 and 24 h post I/R and in sham controls. (*n* = 7 per group). **(F)** Representative images of H&E stained necrotic areas in ischaemic liver lobes from USP10-HZ and WT mice at 6 and 24 h post I/R or from sham control mice (*n* = 7 per group). The results shown are representative of three blots. GAPDH served as the loading control. For statistical analysis, one-way ANOVA was used for panels **(A–D)**, two-tailed Student’s *t*-test was used for panels **(E,F)**. n.s., not significant; *P < 0.05, **P < 0.01.

To analyse whether USP10 is involved in I/R-induced liver dysfunction, the expression of USP10 in the livers of mice subjected to hepatic I/R and primary hepatocytes stimulated by H/R was first measured. In a mouse model of hepatic I/R, the mRNA and protein expression of USP10 was gradually decreased at the indicated time points after reperfusion (Figures 1A,B). Consistently, the expression of USP10 was also significantly gradually reduced in primary hepatocytes at the indicated time points after H/R (Figures 1C,D). These results suggest that USP10 participates in the pathogenesis of hepatic I/R injury.

To investigate the USP10 function in hepatic I/R injury, we constructed USP10 heterozygous mice(USP10-HZ). USP10 knockdown expression was confirmed by western blot analysis (Supplementary Figure 1A). USP10-HZ and WT mice were used to construct a hepatic I/R injury model. USP10 mRNA and protein expression were further reduced in USP10 HZ I/R mice compared with WT I/R mice (Supplementary Figures 1B,C). There were no differences in ALT and AST levels and necrotic area size between the HZ-sham and WT-sham groups. Compared with the WT I/R mice, the levels of ALT and AST in the USP10-HZ group were significantly increased at 6 and 24 h after I/R (Figure 1E). HE staining showed that the necrotic area size in the liver was significantly augmented in the USP10-HZ group at 6 and 24 h (Figure 1F). These results demonstrate that USP10 knockdown aggravates hepatic I/R injury.

### USP10 Knockdown Aggravates Liver Inflammation During Hepatic I/R Injury

**FIGURE 2:**
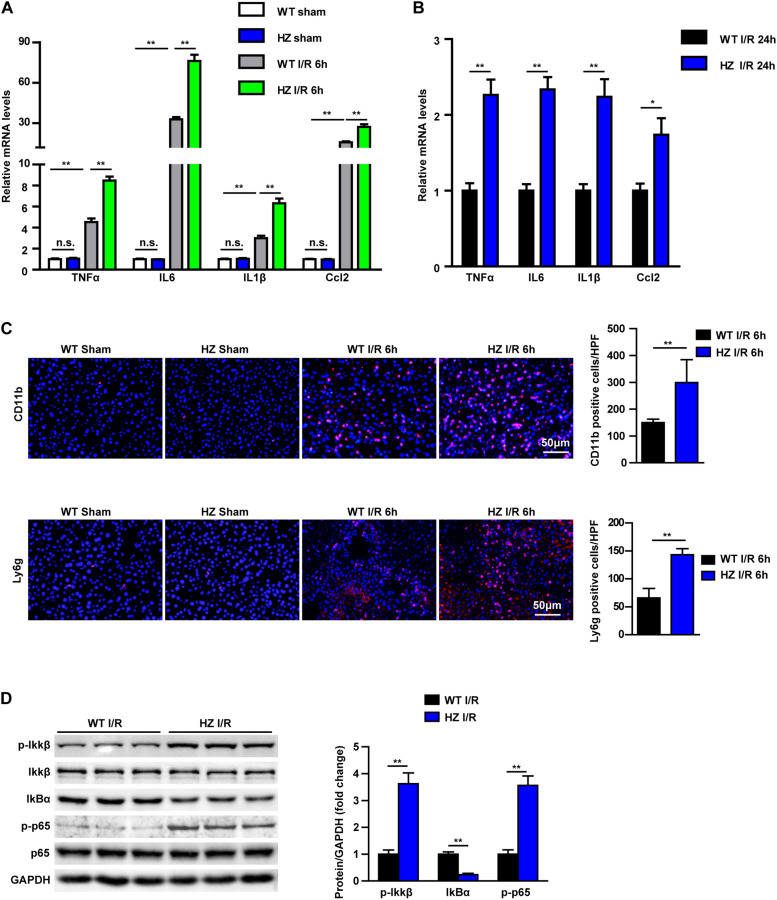
USP10 knockdown enhances the inflammation response during hepatic I/R. **(A,B)** The mRNA level of pro-inflammatory factors (TNF-α, IL6, IL1β, and Ccl2) at 6 h **(A)** and 24 h **(B)** after I/R injury in USP10-HZ and WT mice and in sham controls (*n* = 4 per group). **(C)** The immunofluorescence staining of CD11b and Ly6g in livers of WT and USP10-HZ mice at 6 h post-reperfusion and in sham controls (*n* = 3 per group). **(D)** The protein expression levels of NF-κB signalling components in livers from USP10-HZ and WT mice at 6 h after I/R injury (*n* = 3 per group). GAPDH served as the loading control. For statistical analysis, one-way ANOVA was used for panel **(A)**, and two-tailed Student’s *t*-test was used for panels **(B–D)**. n.s., not significant; *P < 0.05, **P < 0.01.

Inflammation response is the key mechanism of hepatic I/R injury (17). Therefore, we examined the inflammatory response in USP10-HZ and WT mice after I/R and in corresponding sham mice. In the hepatic I/R injury model of USP10-HZ mice, the mRNA expression levels of inflammatory factors in hepatic tissue, such as TNF-α, IL6, IL1β, and Ccl2 were significantly increased after 6 and 24 h of I/R injury (Figures 2A,B). The infiltration of neutrophils (CD11b positive cells and Ly6g positive cells) in hepatic tissue was significantly aggravated (Figure 2C). Furthermore, western blotting showed that the classic pro-inflammatory NF-κB signalling pathway was dramatically upregulated in USP10-HZ group compared with WT mice, demonstrated by the significant increase in p-IKKβ, p-p65, and IκBα degradation (Figure 2D). These observations demonstrate that USP10 knockdown aggravates liver inflammation during hepatic I/R injury.

### USP10 Knockdown Enhanced Hepatocyte Apoptosis During Hepatic I/R Injury

**FIGURE 3:**
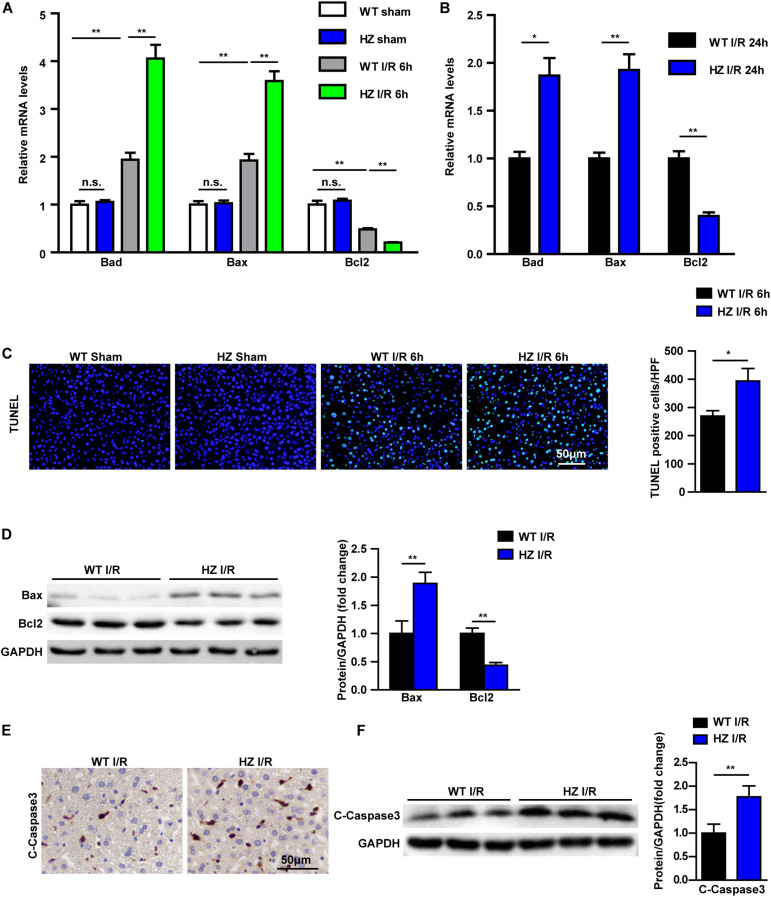
USP10 knockdown promotes apoptosis in hepatic I/R. **(A,B)** The mRNA levels of cell death-related genes (Bad, Bax and Bcl2) in livers from USP10-HZ and WT mice in I/R groups at 6 h **(A)** and 24 h **(B)** post-reperfusion and in sham controls (*n* = 4 per group). **(C)** Representative TUNEL staining in liver lobes from USP10-HZ mice and WT mice in I/R groups at 6 h post-reperfusion and in sham controls (*n* = 3 per group). **(D)** The protein levels of cell death-related genes (Bax and Bcl2) in livers from USP10-HZ and WT mice in I/R the groups at 6 h post-reperfusion (*n* = 3 per group). **(E)** Representative immunohistochemistry staining (C-Caspase3) in liver lobes from USP10-HZ mice and WT mice in I/R groups at 6 h post-reperfusion (*n* = 4 per group). **(F)** The protein levels of C-Caspase3 in liver lobes from USP10-HZ mice and WT mice in I/R groups at 6 h post-reperfusion (*n* = 3 per group). GAPDH served as the loading control. For statistical analysis, one-way ANOVA was used for panel **(A)**, two-tailed Student’s *t*-test was used for panels **(B–F)**. *P < 0.05; **P < 0.01; n.s., not significant.

Apoptosis in USP10-HZ and WT mouse livers was measured after hepatic I/R injury. Real-time reverse transcription-PCR was used to detect the mRNA expression of apoptosis signalling pathway factors. The results showed that the expression of the pro-apoptotic molecules Bax and Bad was increased and the expression of the anti-apoptotic molecule Bcl-2 was significantly decreased in the USP10-HZ group at 6 and 24 h after reperfusion (Figures 3A,B). Terminal deoxynucleotidyl transferase dUTP nick end labelling staining showed that USP10 knockdown dramatically enhanced I/R induced hepatocyte apoptosis (Figure 3C). Moreover, western blot results further validated the downregulation of Bcl-2 and upregulation of Bax in the livers of USP10-HZ mice compared with WT mice at 6 h after hepatic I/R injury (Figure 3D). Immunohistochemical and western blot analysis also showed that USP10 knockdown promoted the I/R induced increase in C-Caspase3 expression (Figures 3E,F). These results demonstrate that USP10 knockdown promotes cell apoptosis during hepatic I/R injury.

### USP10 Overexpression Relieved Hepatic I/R Injury by Inhibiting Hepatocyte Inflammation and Apoptosis

**FIGURE 4:**
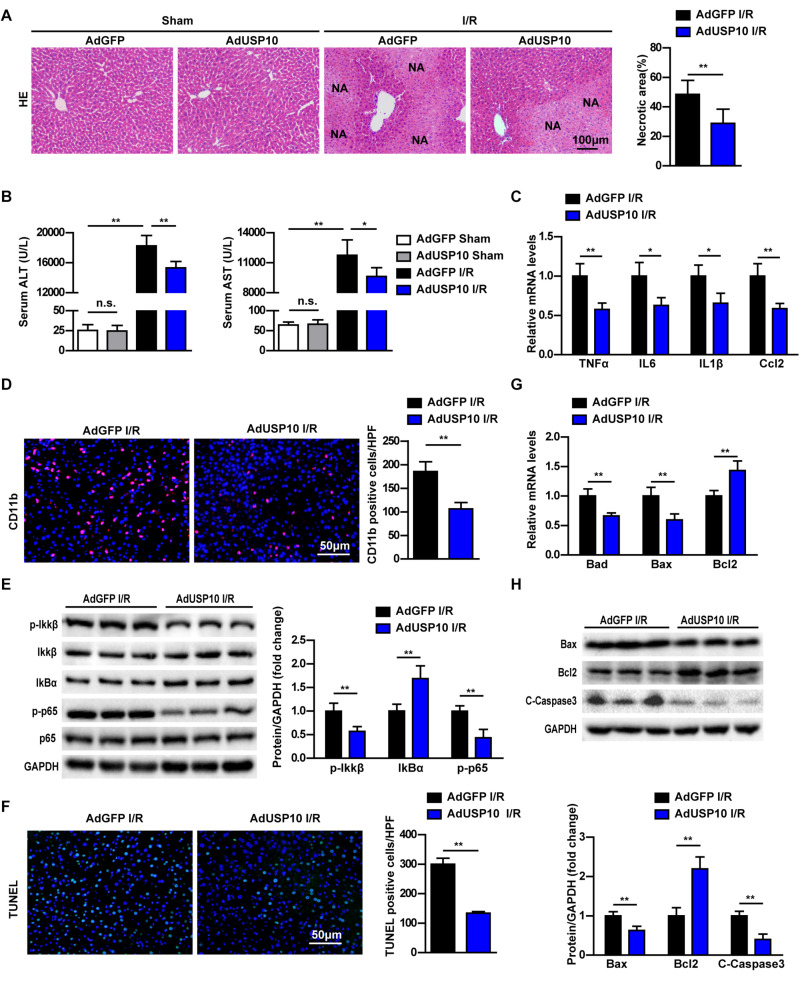
USP10 overexpression relieves hepatic injury by inhibiting inflammation and apoptosis in mice I/R model. **(A)** Representative images of H&E stained necrotic areas in ischaemic liver lobes from AdGFP and AdUSP10 mice at 6 h post I/R or from sham control mice (*n* = 6 per group). **(B)** The serum ALT and AST levels in AdGFP and AdUSP10 mice at 6 h post I/R and in sham controls (*n* = 8 per group). **(C)** The mRNA level of pro-inflammatory factors (TNF-α, IL6, IL1β, and Ccl2) at 6 h after I/R injury in AdGFP and AdUSP10 mice (*n* = 4 per group). **(D)** The immunofluorescence staining of CD11b in livers of AdGFP and AdUSP10 mice at 6 h post-reperfusion (*n* = 3 per group). **(E)** The protein expression levels of NF-κB signalling components in livers from AdGFP and AdUSP10 mice at 6 h after I/R injury. (*n* = 6 per group). **(F)** Representative TUNEL staining in liver lobes from AdGFP and AdUSP10 mice in I/R groups at 6 h post-reperfusion (*n* = 3 per group). **(G)** The mRNA levels of cell death-related genes (Bad, Bax, and Bcl2) in livers from AdGFP and AdUSP10 mice in I/R groups at 6 h post-reperfusion (*n* = 4 per group). **(H)** The protein levels of cell death-related genes (Bax, Bcl2, and C-Caspase3) in livers from AdGFP and AdUSP10 mice in I/R groups at 6 h post-reperfusion (*n* = 6 per group). GAPDH served as the loading control. For statistical analysis, one-way ANOVA was used for panel **(B)**, two-tailed Student’s *t*-test was used for panels **(A,C–H)**. *P < 0.05; **P < 0.01; n.s., not significant.

To further validate this understanding of USP10 function in hepatic I/R injury, the USP10 overexpression adenovirus was constructed and injected into mice to upregulate USP10 expression in liver tissue (Supplementary Figure 1D). Then, the hepatic I/R model was constructed using the same method as described previously. The results showed that USP10 overexpression significantly decreased the levels of serum ALT and AST, and necrotic liver tissue areas size at 6 h after I/R stimulation (Figures 4A,B). The inflammation response and cell apoptosis in AdUSP10 mice were then detected with the same routine. The secretory inflammatory cytokines (TNF-α, IL6, IL1β, and Ccl2) and infiltration of neutrophils (CD11b positive cells) were significantly inhibited (Figures 4C and D). The classic pro-inflammatory NF-κB signalling pathway was dramatically inhibited by USP10 overexpression compared with AdGFP mice (Figure 4E). TUNEL assay showed that USP10 overexpression reduced the number of TUNEL-positive cells in hepatic tissues following I/R stimulation (Figure 4F). Cell apoptosis assay showed that overexpression of USP10 decreased Bad and Bax mRNA expression and upregulated Bcl-2 mRNA expression (Figure 4G). We verified that Bax and C-caspase 3 protein expression was downregulated and Bcl-2 expression was upregulated by Western blot (Figure 4H). The results showed that USP10 overexpression alleviated hepatic I/R injury by inhibiting inflammation and apoptosis.

### USP10 Inhibited Inflammation and Apoptosis in Hepatocytes After H/R Treatment

**FIGURE 5:**
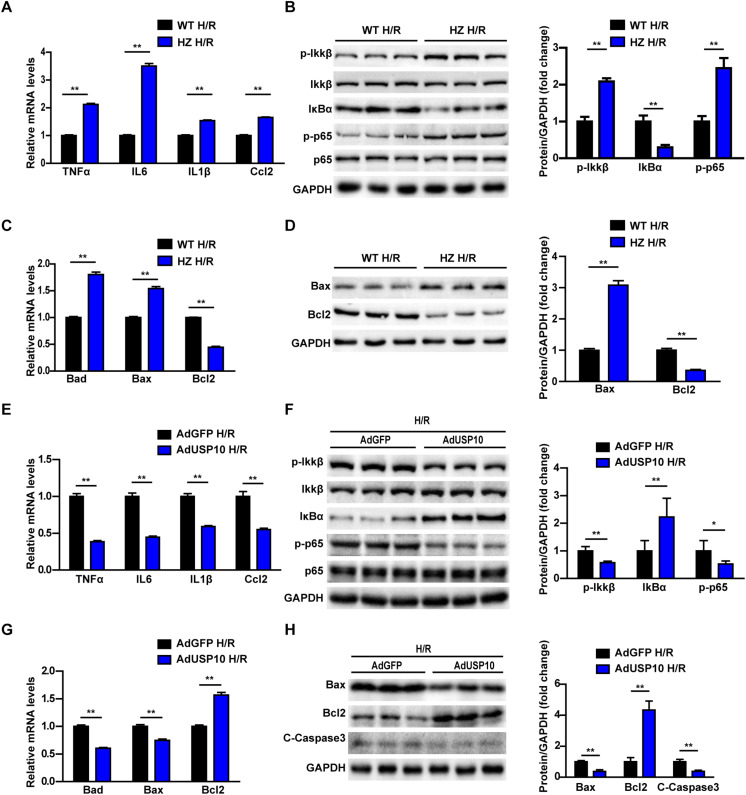
USP10 inhibit inflammation and apoptosis in hepatocytes after H/R treatment. **(A)** The mRNA level of pro-inflammatory factors (TNF-a, IL6, IL1β, and Ccl2) in H/R-treated hepatocytes isolated from USP10-HZ and WT mice. **(B)** The protein expression levels of NF-κB signalling components in the indicated groups. **(C)** The mRNA expression levels of death-related genes (Bad, Bax, and Bcl2) in the indicated groups. **(D)** The protein expression levels of death-related genes (Bax and Bcl2) in the indicated groups. **(E)** The mRNA level of pro-inflammatory factors (TNF-a, IL6, IL1β, and Ccl2) in H/R-treated hepatocytes infected with AdGFP or AdUSP10. **(F)** The protein expression levels of NF-κB signalling components in the indicated groups. **(G)** The mRNA expression levels of death-related genes (Bad, Bax, and Bcl2) in the indicated groups. **(H)** The protein expression levels of death-related genes (Bax, Bcl2, and C-Caspase3) in in the indicated groups. For panels **(B,D,F,H)**, the results shown are representative of three blots, and GAPDH served as the loading control. For statistical analysis, two-tailed Student’s *t*-test was used for panels **(A–H)**, *P < 0.05, **P < 0.01.

To validate whether USP10 directly influences inflammation and apoptosis in hepatocytes, we detected inflammation and apoptosis in H/R-treated primary hepatocytes isolated from USP10-HZ and WT mice. USP10 knockdown in primary hepatocytes significantly promoted the expression of inflammatory factors (TNF-α, IL6, IL1β, and Ccl2) (Figure 5A). The western blot results showed that the classic pro-inflammatory NF-κB signalling pathway was significantly promoted by USP10 knockdown, as demonstrated by the increase in p-IKKβ, p-p65, and IκBα degradation (Figure 5B). Furthermore, qRT-PCR of hepatocytes with H/R from USP10-HZ mice and WT mice demonstrated that the mRNA expression of pro-apoptosis molecules (Bad and Bax) were upregulated and that the anti-apoptotic molecule Bcl-2 was downregulated (Figure 5C). Western blot results further validated the downregulation of Bcl-2 and upregulation of Bax in the hepatocytes from the USP10-HZ group compared with hepatocytes from WT mice at 6 h after H/R stimulation (Figure 5D). Then the hepatocytes with AdUSP10 overexpression were produced by primary hepatocytes infected with AdUSP10. Using the H/R model, we also found that USP10 overexpression significantly inhibited inflammatory cytokine secretion (TNF-α, IL6, IL1β, and Ccl2) and NF-κB activation (Figures 5E,F). Furthermore, the mRNA expression of pro-apoptosis molecules (Bad and Bax) were less and that the expression of anti-apoptotic molecule Bcl-2 was higher in USP10 overexpression group (Figure 5G). Western blot results further validated the upregulation of Bcl-2 levels and downregulation of Bax and C-Caspase3 levels in the AdUSP10-hepatocytes compared with AdGFP-hepatocytes at 6 h after H/R stimulation (Figure 5H). Taken together, these results demonstrated that USP10 inhibited inflammation and apoptosis in hepatocytes after H/R treatment.

#### USP10 Inhibits TAK1-JNK/p38 Activation During Hepatic I/R Injury

**FIGURE 6:**
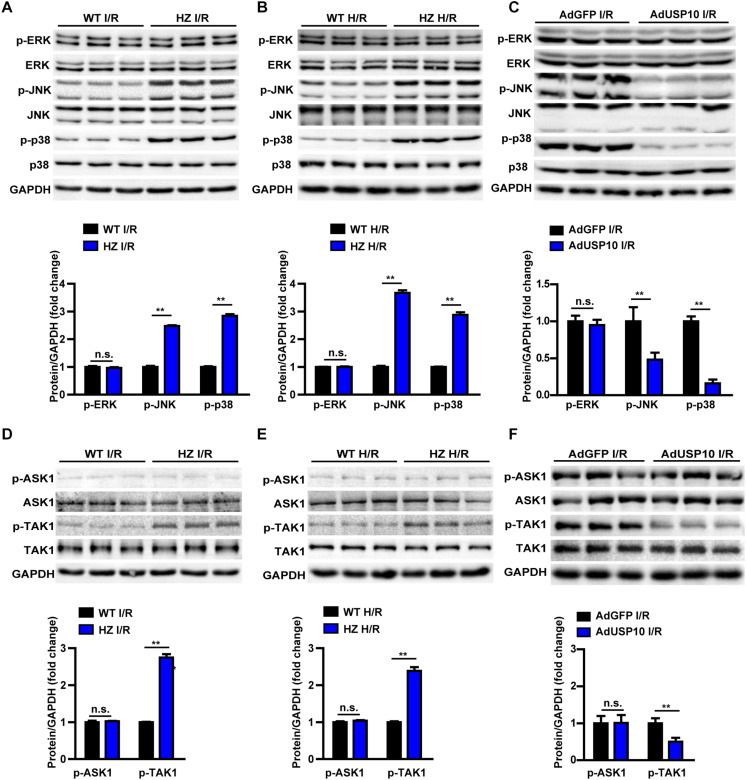
USP10 inhibited TAK1-JNK/p38 signalling in hepatic I/R process. **(A)** Western blots showing the total and phosphorylated ERK, JNK and p38 expression levels in livers from USP10-HZ and WT mice 6 h post-I/R (*n* = 3 per group). **(B)** Western blots showing the total and phosphorylated ERK, JNK and p38 expression levels in H/R-treated hepatocytes isolated from USP10-HZ and WT mice. **(C)** Western blots showing the total and phosphorylated ERK, JNK and p38 expression levels in livers from AdGFP and AdUSP10 mice at 6 h post-I/R injury (*n* = per group) **(D)** Western blots showing the total and phosphorylated TAK1 and ASK1 expression levels in livers from USP10-HZ and WT mice 6 h post-I/R injury (*n* = 3 per group). **(E)** Western blots showing the total and phosphorylated TAK1 and ASK1 expression levels in H/R-treated hepatocytes isolated from USP10-HZ and WT mice. **(F)** Western blots showing the total and phosphorylated TAK1 and ASK1 expression levels in livers from AdGFP and AdUSP10 mice 6 h post-I/R injury (*n* = per group). The results shown are representative of three independent experiments. GAPDH served as the loading control. For statistical analysis, two-tailed Student’s *t*-test was used for panels **(A–F)**. n.s., not significant; **P < 0.01.

MAPKs play an important role in mediating the inflammatory response and cell death. Many studies have shown that I/R surgery or H/R injury activates MAPK signalling, as evidenced by phosphorylation of JNK (p-JNK) and ERK (p-ERK) in the liver and primary hepatocytes (18, 19). Therefore, we investigated whether USP10 deficiency promotes hepatic I/R injury in a MAPK-dependent manner. However, in our experiment, we found that USP10 knockdown only increased p-JNK and p-p38 levels but not p-ERK levels in liver after I/R injury or in hepatocytes after H/R treatment (Figures 6A,B). Furthermore, we detected that the MAPK signalling pathway in AdUSP10 mice stimulated with I/R. The results showed that USP10 overexpression inhibited the liver tissue JNK/P38 activation after I/R injury (Figure 6C). The phosphorylation of TAK1 (p-TAK1) and ASK1 (p-ASK1) often regulate MAPKs in multiple physiological processes. Thus, we examined the activation of p-ASK1 and p-TAK1, which are well understood upstream factors of the JNK and p38 signalling pathways. We also found that TAK1 activation, but not ASK1 activation, was responsive to USP10 expression changes *in vivo* and *in vitro* (Figures 6D–F). Together, these observations suggest that USP10 inhibited the activation of TAK1-JNK/p38 signalling during hepatic I/R injury.

### TAK1 Mediates the Detrimental Effect of USP10 During Hepatic I/R Injury

**FIGURE 7:**
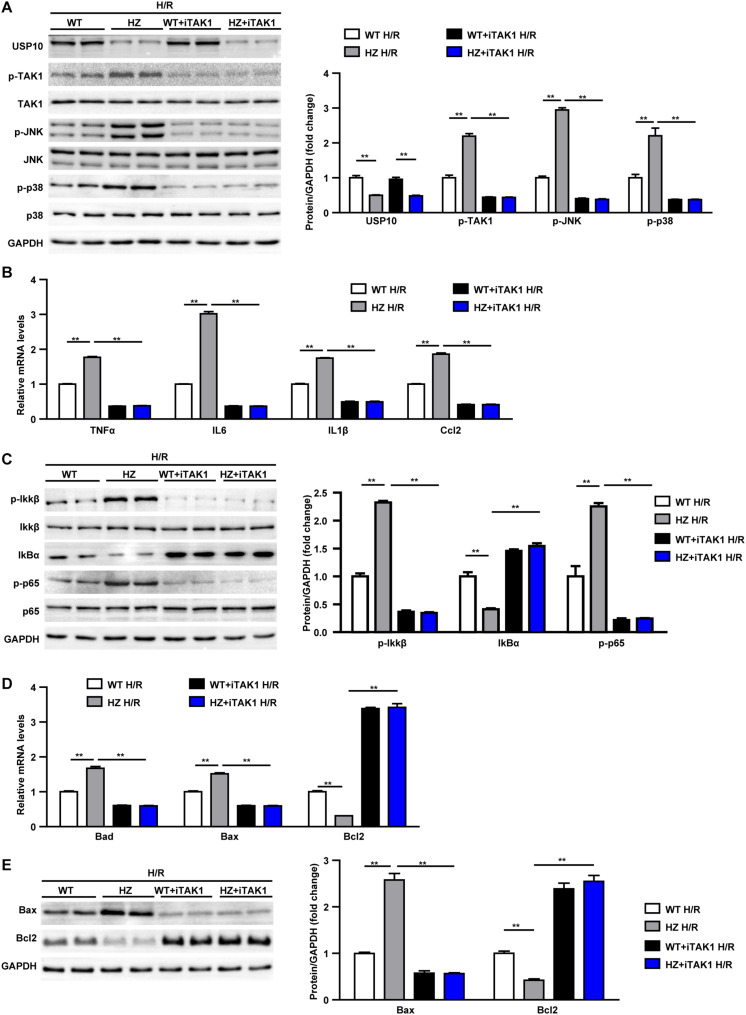
TAK1 inhibitor treatment blocked the detrimental effect of UPS10 knockdown on hepatocyte stimulated with H/R. **(A)** Protein levels of USP10, total and phosphorylated TAK1, JNK and p38 in hepatocytes from USP10-HZ and WT mice treated with vehicle or TAK1 inhibitor and harvested at 6 h after H/R. **(B)** mRNA levels of pro-inflammatory factors (TNF-α, IL6, IL1β, and Ccl2) in hepatocytes from the indicated groups. **(C)** Protein levels of NF-κB signal pathway components in hepatocytes from the indicated groups. **(D)** mRNA levels of cell death-related genes (Bad, Bax, and Bcl2) in hepatocytes from the indicated groups **(E)** Protein levels of cell death-related genes (Bax and Bcl2) in hepatocytes from the indicated groups. For panels **(A,C,E)**, the results shown are representative of three blots, and GAPDH served as the loading control. For panels **(B,D)**, the results shown are representative of three independent experiments. For statistical analysis, one-way ANOVA was used for panels **(A–E)**. **P < 0.01.

**FIGURE 8:**
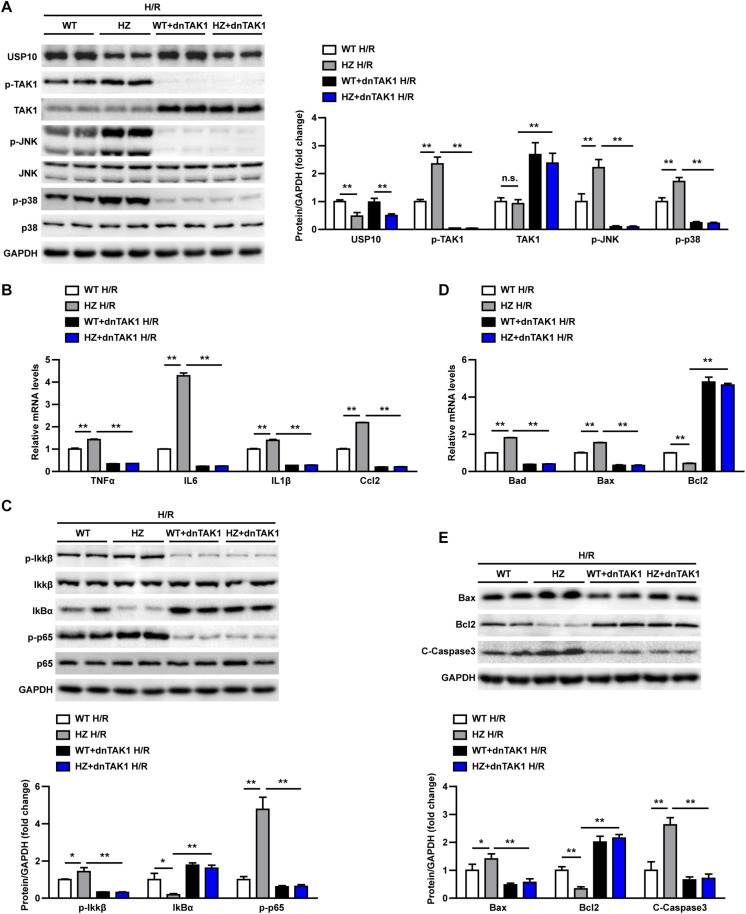
TAK1 dominant negative mutant adenovirus(dnTAK1) transduction reversed the detrimental effect of UPS10 knockdown on hepatocyte stimulated with H/R. **(A)** Protein levels of USP10, total and phosphorylated TAK1, JNK, and p38 in hepatocytes isolated from USP10-HZ and WT mice treated with dnTAK1 or AdGFP and harvested at 6 h after H/R. **(B)** mRNA levels of pro-inflammatory factors (TNF-α, IL6, IL1β, and Ccl2) in hepatocytes from the indicated groups. **(C)** Protein levels of NF-κB signal pathway components in hepatocytes from the indicated groups. **(D)** mRNA levels of cell death-related genes (Bad, Bax, and Bcl2) from the indicated groups. **(E)** Protein levels of cell death-related genes (Bax, Bcl2, and C-Caspase3) in hepatocytes from the indicated groups. For panels **(A,C,E)**, the results shown are representative of three blots, and GAPDH served as the loading control. For panels **(B,D)**, the results shown are representative of three independent experiments. For statistical analysis, one-way ANOVA was used for panels **(A–E)**. n.s., not significant; *P < 0.05, **P < 0.01.

In the last stage, we evaluated whether USP10 regulates hepatocyte inflammation and apoptosis was dependent on TAK1. Primary hepatocytes were isolated from the livers of USP10-HZ and WT mice. The TAK1 inhibitor 5Z-7-ox was used to block the activity of TAK1 and its downstream signalling pathway molecules p38 and JNK (Figure 7A). The results showed that USP10 knockdown significantly increased the expression of inflammatory factors (TNF-α, IL6, IL1β, and Ccl2) and activation of NF-κB signalling in hepatocytes after H/R treatment; however, this effect was blocked by inhibition of TAK1 (Figures 7B–C). Notably, TAK1 inhibition significantly protected against hepatocyte apoptosis aggravated by USP10 knockdown, as evidenced by decreased mRNA and protein expression of Bax and increased mRNA and protein expression of Bcl2 (Figures 7D,E). To avoid the unspecific TAK1 inhibitor effect, TAK1 dominant negative mutant adenovirus was produced to completely inhibit TAK1 function (Figure 8A), which was then stimulated with H/R at 6 h. The results showed that dnTAK1 protected against hepatocyte inflammation and apoptosis aggravated by USP10 knockdown during the H/R process (Figures 8A–E).

These observations demonstrated that TAK1 inhibition abolished the effect of USP10 knockdown on inflammation and hepatocyte apoptosis, suggesting that TAK1 mediates the protective function of USP10 in hepatic I/R injury.

## Discussion

USP10 is a deubiquitinating enzyme and a critical factor in controlling protein aggregation, aggresome formation, and cytotoxicity in protein aggregation-related diseases (6). USP10 interacts with the ubiquitin receptor p62 and the interaction augments p62-dependent ubiquitinated protein aggregation and aggresome formation, thereby cooperatively inhibiting apoptosis (20). However, the role of USP10 in hepatic I/R injury has not been reported. In the present study, we detected that during the development of hepatic I/R in mice and hepatocytes, USP10 expression was downregulated, indicating that USP10 may play an important role in the progression of hepatic I/R injury. Based on various *in vivo* and *in vitro* hepatic I/R models, we clearly demonstrated that USP10 alleviated hepatic I/R injury via inhibition of inflammation and apoptosis. Mechanistically, USP10 inhibited the activation of TAK1-JNK/p38 signalling, and the detrimental role of USP10 deficiency in hepatic I/R injury was blocked by inhibition of TAK1 activation in hepatocytes. Collectively, this study reveals a novel function of USP10 in hepatic I/R injury and suggests that USP10 may serve as a potential target to alleviate the pathological process of hepatic I/R injury.

More and more studies have shown that sterile inflammation and apoptosis play an important role in hepatic ischemic reperfusion. During periods of liver ischaemia, glycogen consumption and energy depletion lead to hepatocyte apoptosis or even necrosis. The apoptosis/necrotic cells release HMGB1 and other DAMPs. These danger signals recruit inflammatory cells into liver tissue during reperfusion and release inflammatory factors, which cause further apoptosis and necrosis in hepatocytes. NF-κB,MAPK, and other signalling pathways play an important role in the inflammatory response of hepatic ischaemia reperfusion (21). Many studies have shown that USP10 is a key molecule in regulating inflammation and apoptosis through various pathways (6, 22, 23). For example, USP10 promotes proliferation and migration and inhibits apoptosis of endometrial stromal cells in endometriosis by activating the Raf-1/MEK/ERK pathway (24). In hepatocellular cancer, USP10, as a tumour suppressor, negatively regulates mTORC1 activation and AKT phosphorylation by stabilising AMPKα and PTEN in HCC cells (13). USP10 is also a regulator of the EMT-transcription factor SLUG and of cell migration (25). In the NFALD model, USP10 regulates hepatic steatosis by interacting with SIRT6 and inhibiting its ubiquitination and degradation. SIRT6 overexpression markedly ameliorated the effects of USP10 deficiency on hepatic steatosis, insulin resistance, and inflammation (6). In addition, AMP-activated protein kinase (AMPK) is the master regulator of metabolic homoeostasis by sensing cellular energy status. USP10 and AMPK can form a key feedforward loop that ensures amplification of AMPK activation in response to fluctuations in cellular energy status (26). In fact, USP10 knockdown mice exhibited more severe inflammatory responses and aggravated apoptosis via the NF-κB pathway (Figures 1–3). We also demonstrated from the opposite perspective that USP10 overexpression alleviates liver I/R injury by inhibiting hepatocyte inflammation and apoptosis (Figure 4).

At the cellular level, the NF-κB pathway is the major pathological link among inflammation in hepatic I/R injury (27). A previous study demonstrated that USP10 inhibits NF-κB activation and inflammatory cytokine induction (22). Consistently, in our study, we also demonstrated that USP10 inhibited hepatocyte inflammation and apoptosis in hepatocytes following H/R stimulation (Figure 5).

In hepatic I/R, ischaemia induces the decline of ATP and increases the ratio of AMP/ATP, which promotes the phosphorylation and activation of AMPK. Three AMPK kinases, liver kinase B1 (LKB1), Ca2+/calmodulin-dependent protein kinase kinase β (CaMKKβ), and TAK1, are the main upstream kinases for the phosphorylation of AMPK (28, 29). TGF-β-activated kinase-1 is essential for TNF-α-mediated activation of NF-κB, JNK, and p38. Previous studies have shown that TAK1 regulates inflammation in liver ischaemia reperfusion (14). Inhibition of TAK1 phosphorylation and MAPK signalling activation can protect against cell death and inflammation during hepatic I/R injury (4, 14, 30). In this study, we also demonstrated that USP10 inhibited hepatocyte inflammation and apoptosis, depending on TAK1 phosphorylation, and JNK and p38 activation (Figure 6). To further confirm the role of TAK1 in the regulation of I/R injury by USP10 knockdown, we blocked TAK1 functions through a TAK1 inhibitor or TAK1 dominant negative mutant adenovirus in primary hepatocytes. When TAK1 was inhibited, the effect of USP10 knockdown on hepatocyte damage was reversed, indicating that USP10 protects against hepatic I/R injury by TAK1 activation (Figures 7, 8). Previous studies have shown that USP10 can play a role in inflammation and apoptosis via ubiquitination of various substrate proteins, including TAK1 (5, 6, 31).

In conclusion, the current study showed that USP10 is an essential molecule in the development of hepatic I/R, and the effects of USP10 are dependent on the TAK1-JNK/p38 pathway. Thus, targeting USP10 may represent a promising strategy to alleviate hepatic I/R injury.

## Data Availability Statement

The datasets generated for this study are available on request to the corresponding author.

## Ethics Statement

The animal study was reviewed and approved by Renmin Hospital of Wuhan University.

## Author Contributions

QT and ZJQ designed the study and performed the research. WTY performed the experiments. CZB and MX contributed essential reagents or tools. ZL and ZJL analysed the data and performed statistical analysis. QT wrote the manuscript. All authors contributed to the article and approved the submitted version.

## Conflict of Interest

The authors declare that the research was conducted in the absence of any commercial or financial relationships that could be construed as a potential conflict of interest.

## Supplementary Material

Supplementary Figure 1 USP10 expression identification in USP10-HZ mice and AdUSP10 mice and USP10 expression in I/R model. **(A)** USP10 protein expression in livers from USP10-HZ and WT mice (n = 3 per group). **(B,C)** USP10 protein and mRNA expression in livers from USP10-HZ and WT mice stimulated with I/R 6 h or sham treatment (n = 3 per group). **(D)** USP10 protein expression in livers from mice treated with adenovirus GFP(AdGFP) or adenovirus USP10(AdUSP10). GAPDH served as the loading control. For statistical analysis, two-tailed Student’s t-test was used for B-D, ^∗∗^*P* < 0.01.

## Abbreviations

ALT: alanine aminotransferase
AST: aspartate aminotransferase
AdUSP10: Adenovirus USP10
DAMP: danger-associated molecular patterns;DnTAK1,domain negative TAK1
DMEM: Dulbecco’s Modified Eagle’s Medium
H&E: haematoxylin and eosin
HZ: heterozygous
I/R: ischaemic/reperfusion
LT: liver transplantation
mRNA: messenger RNA
NF- κ B: nuclear factor kappa B
NAFLD: non-alcoholic fatty liver disease
PCR: polymerase chain reaction
TUNEL: Terminal deoxynucleotidyl transferase dUTP nick end labelling
USP10: ubiquitin-specific peptidase 10
TAK1: transforming growth factor beta-activated kinase 1
WT: wild type.
