# Supplementary figures and images for: Ubiquitin-Specific Peptidase 10 Protects Against Hepatic Ischaemic/Reperfusion Injury via TAK1 Signalling
Source: Front Immunol. 2020 Sep 29;11:506275. doi: 10.3389/fimmu.2020.506275 (PMC7550542; doi:10.3389/fimmu.2020.506275)

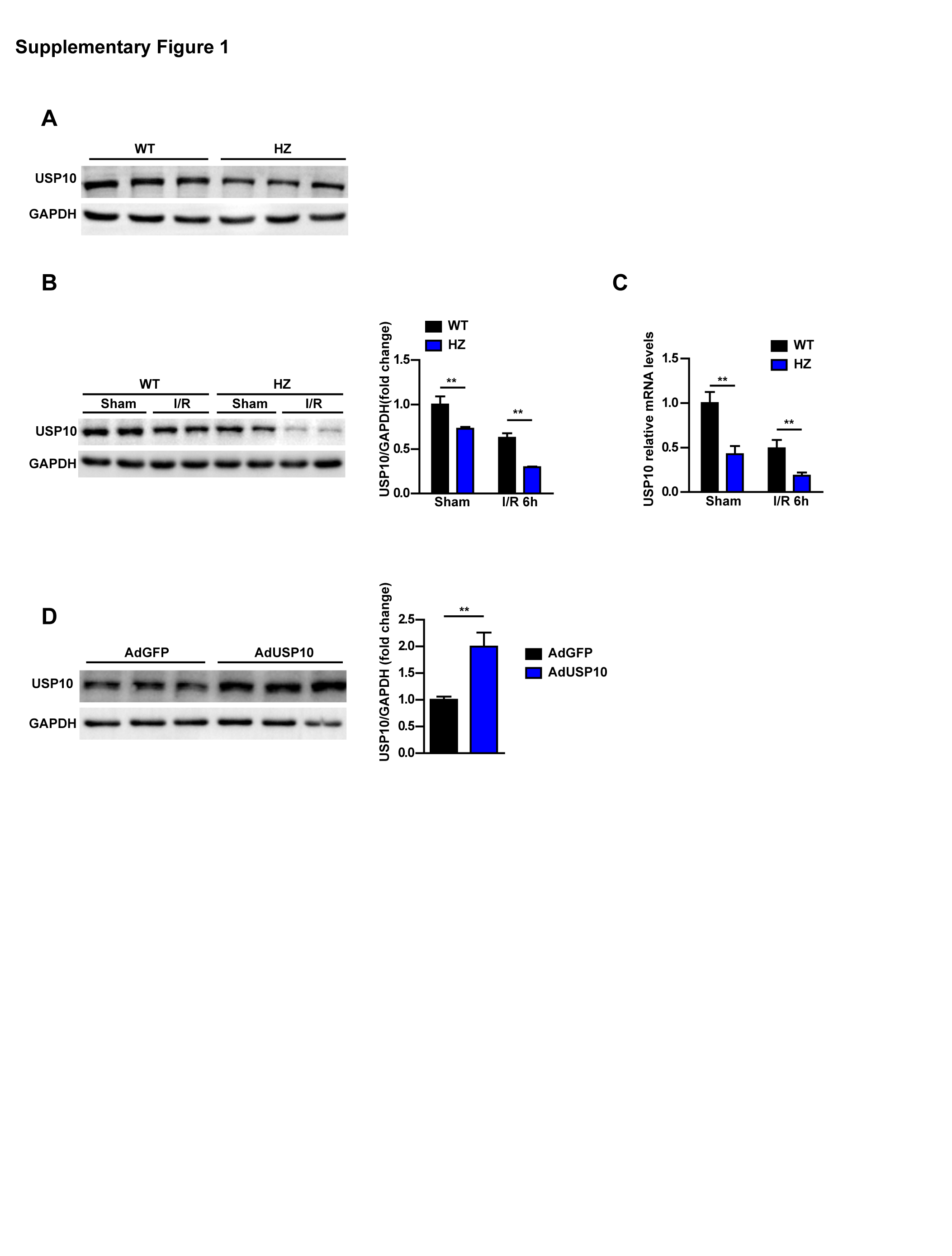

Supplement: Supplementary Figure 1 — USP10 expression identification in USP10-HZ mice and AdUSP10 mice and USP10 expression in I/R model. (A) USP10 protein expression in livers from USP10-HZ and WT mice (n = 3 per group). (B,C) USP10 protein and mRNA expression in livers from USP10-HZ and WT mice stimulated with I/R 6 h or sham treatment (n = 3 per group). (D) USP10 protein expression in livers from mice treated with adenovirus GFP(AdGFP) or adenovirus USP10(AdUSP10). GAPDH served as the loading control. For statistical analysis, two-tailed Student’s t-test was used for B-D, ∗∗P < 0.01. [file Image_1.tif]
